# Some Advice of a Practical Nature

**Published:** 1920-08-28

**Authors:** 


### EARLY SYMPTOMS AND SIGNS OF NERVOUS DISEASE.

### Some Advice of a Practical Nature.

At the recent British Medical Association meeting, the section of neurology and psychiatry, under the presidency of Dr. Henry Head, had a stimulating discussion on early symptoms and signs of nervous disease and their interpretation. An opening speech, by force of circumstance, is rarely a complete *résumé* of the subject with which it deals, but on this occasion the President touched so frequently upon that aspect of nervous disease which is the very keynote of the practitioner's view of these cases that we feel his advice may be of exceptional use and interest. Dr. Head, upon whose address these notes are substantially based, explained that it was the hope of those who had suggested the discussion that the combined experience of the meeting would show which of those early signs and symptoms were of diagnostic and prognostic value. Early signs (he pointed out) are not only neglected because they seem trivial, but the physician frequently omits to establish their true nature during the short period of their existence, and, on the other hand, any morbid condition with an acute or fulminating onset is liable to be accompanied by signs and symptoms far in excess of the structural changes on which it is based. All disease of the nervous system manifests itself in loss of function. Not only does the patient complain of some lack of functional capacity, but the physician has to arrange his tests to elicit such defects.

### The Word "Functional."

In his view a strong protest should be made against the perverted use of the word "functional." Many employ it as an euphemism for "hysterical," and the subtle effect of this false use of the word must sooner or later corrupt their diagnostic thinking. Some condition for which they cannot determine a cause is labelled "functional," and treatment carried out on the negligent and irrational lines usually considered suitable for hysteria. An even more insidious danger in the careless use of this work may arise from an antithesis in the physician's mind between "functional" and "organic." As far as the nervous system is concerned, morbid conditions can express themselves solely through the disturbance of function. The grossest destruction may be evident in some paralysis or overaction only. But experience has associated certain signs with destruction of certain parts of the nervous system, and these are spoken of by prolepsis as "organic" signs; whereas they are, in reality, evidence of disturbed function and not of structural changes.

### The Personality of the Patient.

Later Dr. Head indicated some of the earliest changes associated with disease of the central nervous system. With him we realise that one of these is an undoubted alteration in the personality of the patient. Cerebro-spinal syphilis may bring about such a change in character as to make the previously efficient workman not worth his pay. Frontal tumours are notoriously liable to change the personality. An instance is given of a modest and retiring Nonconformist minister, who was in hospital for observation, cracked foolish jokes with the nurses, and insisted on accompanying the matron through the wards, clad in dressing-gown and slippers! The disproportionate cheeriness associated in some instances with disseminated sclerosis, is less often recognised and escapes notice because of the insidiousness of the change. Memory may be profoundly affected; sleep disturbed with the onset of many nervous affections, and changes in articulation, again, are amongst the earliest symptoms, but it is remarkable how often these pass unnoticed by those in close contact with the patient. Headache is sometimes treated as if all pains in the head were of similar diagnostic import. The so-called "neurasthenic" headache is present in the morning on waking, and passes off during the day, but it is liable to return at night, evoked by anything that causes fatigue, bodily or intellectual.

### A Threefold Process.

What actually constitutes a disease of the nervous system? asked Dr. Head. Diagnosis to be complete must be a threefold process. First, by careful examination, the nature and extent of the loss of function which constitutes the morbid condition must be elucidated. Next, these symptoms and signs must be translated into terms of some local affection. Finally, if possible, the nature of the underlying pathological process must be discovered. For example, all such names as "tabes dorsalis" and "general paralysis" should be avoided or employed solely as a part of the full diagnosis. They are, in reality, nothing but shorthand titles for groups of signs and symptoms evoked by the action of the spirochæta pallida on different structures of the central nervous system.

### The Real Idea.

Dr. Head said that he would never forget hearing Dr. Hughlings Jackson say on one occasion that when a patient came to him with a pain in his chest and declared that he "felt as if his heart was bursting and as if he was going to die," he (Jackson) would say to him, "And have you ever died? Can you tell me what it feels like to die?" Jackson was not laughing at the patient; he seriously wanted to get at the back of the expressions which the patient used. In the same way, he urges, must we try to get at the real idea of the patients when they say, as they frequently do, that they feel as if something had happened inside the head, or as if their brains had turned over. These expressions, often adopted by the patients, are labels for something they cannot explain, but register very often a change in the affective state, and, as diagnosis indicates, are of the greatest potential value.

